# Amino- and Thiol- Polysilsesquioxane Simultaneously Coating on Poly(*p*-Phenylenetherephthal Amide) Fibers: Bifunctional Adsorbents for Hg(II)

**DOI:** 10.3389/fchem.2019.00465

**Published:** 2019-07-02

**Authors:** Yunfeng Wang, Rongjun Qu, Yinglei Mu, Changmei Sun, Chunnuan Ji, Ying Zhang, Kai An, Xinhua Jia, Yu Zhang

**Affiliations:** School of Chemistry and Materials Science, Ludong University, Yantai, China

**Keywords:** polysilsesquioxane, poly(*p*-phenylenetherephthal amide), bifunctional adsorbent, adsorption, Hg(II)

## Abstract

A double reagents simultaneous functionalization (DRSF) was used to prepare porous polysilsesquioxane with NH_2_ and SH bifunctional groups (PAMPSQ) coated poly(*p*-phenylenetherephthal amide) (PPTA) fibers adsorbents (PPTA-AM), via *in situ* condensations with aminopropyltriethoxysilane (APTES) and mercaptopropyltriethoxysilane (MPTES). The PAMPSQ coated on the PPTA surface was in the form of nanoparticles and its morphology varied with the proportion of the reactants. The PAMPSQ exhibited loose open meso- or macroporous features. The functional groups utilization of PAMPSQ was much higher than those of polysilsesquioxane on the mono-functional adsorbents with thiol or amino groups. The selective adsorption of PPTA-AM adsorbents for Hg(II) in binary component metal ion systems indicated their potential application in environmental remediation. The adsorption mechanism of Hg(II) onto PPTA-AM was proposed.

## Introduction

Water contamination caused by heavy metal ions has become a serious worldwide environmental problem that threatens the ecosystem, food safety, and human health. Thus far, many technologies such as adsorption, solvent extraction, ion exchange, reverse osmosis, membrane filtration, chemical precipitation, and electrolysis have been developed to remove heavy metal ions from contaminated water samples (Huang et al., 1996; Basso et al., 2002; Gomez-Salazar et al., 2003). Among them, adsorption is considered to be the optimum choice because it is facile and highly effective (Zub et al., 2005; Li et al., 2011).

One particular type of adsorbents is based on polysilsesquioxanes, which have desirable properties such as good hydrophilicity, chemical stability, and thermal stability (Hua et al., 2012; Sun et al., 2014). However, there are also unresolved issues regarding easy agglomerate and low utilization rate of the functional groups that eventually limit their applications (Liu et al., 2011). Attempts were made by our group to solve some of these issues. Two types of mono-functionalized fibrous adsorbents were prepared via the sol-gel condensation reactions (Wang et al., 2017). PPTA fibers were separately coated with amino-polysilsesquioxane and thiol-polysilsesquioxane. The resulting adsorbents, denoted as PPTA-A and PPTA-M, respectively, showed enhanced adsorption capacity for Hg(II) as compared to common silica adsorbents and amino- or thiol- functionalized polysilsesquioxane alone, due to the increase in specific surface area and functional group utilization rate. In principle, adsorption capacity of this type of functionalized adsorbents depends on the affinity of the functional group to the metal ion, as well as the morphology of the adsorbent material which affects the functional group utilization rate. PPTA-A is supposed to have similar or slightly better adsorption capacity compared to PPTA-M because of the superior hydrophilicity of the amino group vs. the thiol group. However, PPTA-A exhibited compact gel structures due to existence of lots of hydrogen bond while PPTA-M was able to form loose meso- or macro-porous structures under similar conditions. The overall effect of these two contradicting factors was that the adsorption capacities of PPTA-A samples were much lower than those of PPTA-M ones. It is our assumption that the strong hydrogen bonds between amino groups and PPTA fibers resulted in the compact structures, while the lack of such interaction between thiol groups and PPTA fibers led to the formation of meso- or macro-porous structures. But problems still remain, as the utilization ratio of SH in PPTA-M is unsatisfactory.

In current work, in order to obtain a more ideal adsorbent owning amino and thiol groups at the same time, amino-polysilsesquioxane, and thiol-polysilsesquioxane were introduced onto PPTA by a process named double reagents simultaneous functionalization (DRSF). The combination of weak alkaline amino groups and weak acidic thiol groups, on one hand can reduce the hydrogen bond interactions between the PPTA and functional groups then form porous structures on the surface of PPTA; and introduce simultaneously two kinds of functional groups only via one step on the other. Thus, the adsorbents might enhance Hg(II) separation effect and the utilization of functional groups. The synthesis proportions of amino groups and thiol groups were optimized. The adsorption kinetics, isotherms, selectivity, and adsorption mechanism of the resulting bi-functionalized adsorbent were investigated and discussed. This novel adsorbent showed favorable pore structures and enhanced adsorption capacities.

## Experimental

### Materials and Characterization Methods

PPTA were provided by Yantai Tayho Advanced Materials Co. Ltd., China. 3-Amino-propyltriethoxysilane (APTES) and 3-mercaptopropyltriethoxysilane (MPTES) were bought from Qufu Wanda Chemical Industry Co. Ltd., China. Dimethylsulfoxide (DMSO) and sodium hydride (NaH) were provided from Kishida Chemicals (Tokyo, Japan). Other reagents and solvents were all of analytical grade and were used as received directly.

Infrared (IR) spectra were measured on a fourier transform infrared (FTIR) spectrophotometer Nicolet iS50 (Nicolet, American). Surface morphologies were examined using Field Emission Scanning Electron Microscope (FE-SEM SU8010) (Hitachi, Japan). Thermogravimetric analysis (TGA) was analyzed on a TA instrument for thermogravimetric analysis (NETZSCHSTA 409 thermal analyzer, Germany). Elemental analysis was obtained using an Elementar Vario EL b model elemental analyzer (Elementar, Germany). Wide-angle X-ray diffraction (WAXD) curves were carried out on a Rigaku-D/max-2500VPC (Japan). X-ray photoelectron spectroscopy (XPS) was performed on ESCALAB Xi^+^ (Thermo Fisher Scientific, American). The parameters of the porous structures were determined using an automatic physisorption analyzer (ASAP 2020, Micromeritics, USA). Analysis of various metal ions was performed on a flame atomic absorption spectrophotometer (Varian AA240, American). The contents of -NH_2_ and -SH groups were determined by elemental analysis.

### Preparation of PPTA-AM

**Scheme 1:**
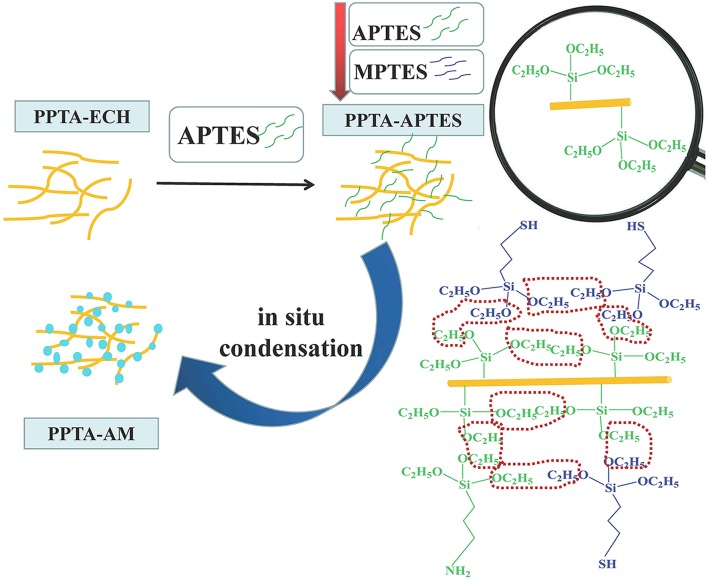
Schematic diagram of the simultaneous functionalization process for PPTA-AM.

Preparation of PPTA-ECH and PPTA-APTES followed the method in our previous work (Xu et al., 2016; Wang et al., 2017). Then different molar ratios of APTES and MPTES (see Table 1) were dissolved in 150 mL DMSO and added to PPTA-APTES in the flask. The mixture was subsequently stirred at 60°C for 12 h and was cooled to room temperature. NH_4_F of 4 mL (0.014 g mL^−1^) was added gradually with stirring and the resulting mixture was stirred for an additional 24 h. Solid fibers and solution were then poured into a Teflon-lined reactor and let to stand for 7 days, maintained at a near-constant temperature of 80°C. Finally, the resulted fibers were separated from the solution, extracted using re-fluxing ethanol for 48 h and dried under vacuum at 60°C for 80 h. The final products were denoted as PPTA-AM-n as shown in Table 1, where n corresponds to the percentage of APTES added. The synthetic route for the preparation of PPTA-AM samples is illustrated in Scheme 1.

**Table 1:** Formulations, element concentrations and binding energies of N_1s_, S_2p_, and Si_2P_ of PPTA and PPTA-AM samples.

Samples	APTES (mol)/ MPTES (mol) (Molar ratio)	Element concentrations Atomic %					Functional groups content (mmol g^−1^)	
		C_1S_	N_1S_	O_1S_	Si_2P_	S_2P_	NH_2_	SH
PPTA	0/0	73.68	7.85	19.47				
PPTA-AM-90	136.17/15.13 (9/1)	67.14	6.07	16.37	8.63	1.79	1.25	0.55
PPTA-AM-70	105.91/45.39 (7/3)	58.26	5.94	19.52	11.91	4.37	0.95	0.97
PPTA-AM-50	75.65/75.65 (1/1)	53.35	5.61	23.74	11.97	5.33	0.73	1.27
PPTA-AM-30	45.39/105.91 (3/7)	52.04	5.26	22.49	13.30	6.91	0.42	1.58
PPTA-AM-10	15.13/136.17 (1/9)	51.75	5.06	22.16	13.19	7.84	0.13	1.75

### Adsorption Experiments

The adsorption experiments were carried out using 20 mL of different concentrations of Hg(II) solution with predetermined amounts of PPTA-AM. The mixture solution was shaken at 25°C and pH 5.0 for 24 h. The equilibrium concentration of each solution was measured by Varian AA240. The adsorption capacity of Hg(II) was calculated according to Equation (1):

(1) $$\begin{array}{l} q_{e}=\frac{\left(C_{0}-C\right)V}{W} \end{array}$$

where *q*_e_ represents the adsorption amount (mmol g^−1^); *C*_0_ and C represent the initial and final concentrations, respectively (mmol mL^−1^); *V*, the volume of solution (mL); *W*, the weight of adsorbents (g).

Adsorption kinetics were investigated from 0 to 7 h and the amount of adsorbent used was 20 mg. In addition, the effect of Hg(II) initial concentration was also studied. The initial concentrations were varied from 1 to 5 mmol L^−1^ at 25°C.

Adsorption selectivity of PPTA-AM-n was established by analyzing a solution containing Hg(II) and a coexisting ion including Cu(II), Ni(II), Pb(II), Ag(I), and Cd(II). The used adsorbents, PPTA-AM-n with adsorbed Hg(II), were eluted using different percentages (0, 1, 2, 3, 4, and 5%) of thiourea in 0.5 M HCl. The most effective eluent of 4% thiourea in 0.5 M HCl was used in five adsorption-desorption cycles on each adsorbent sample.

## Results and Discussion

### Characterization of PPTA-AM

#### IR Spectroscopy Analysis

**Figure 1:**
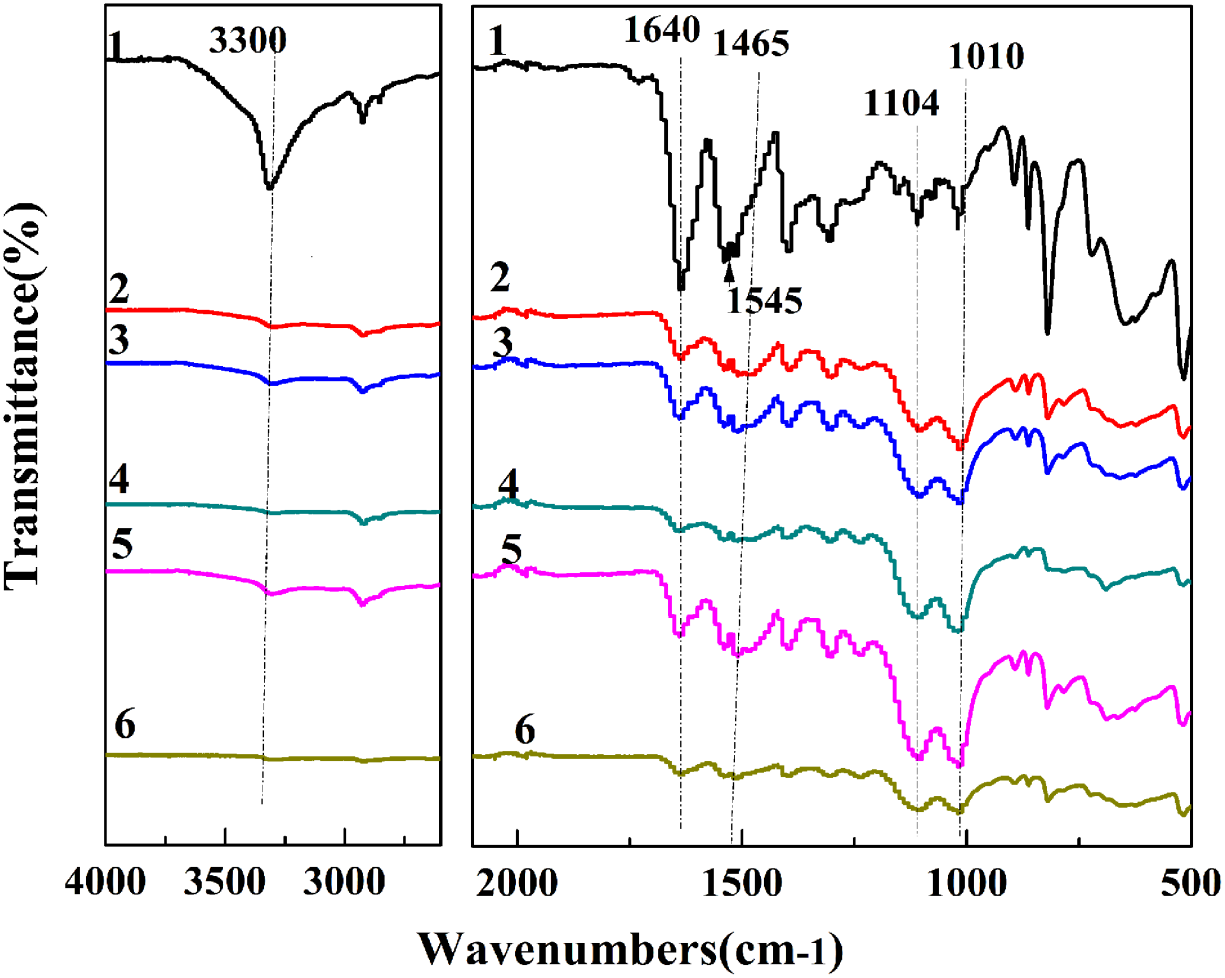
IR spectra of PPTA and PPTA-AM samples. PPTA (1), PPTA-AM-90 (2), PPTA-AM-70 (3), PPTA-AM-50 (4), PPTA-AM-30 (5), and PPTA-AM-10 (6).

The IR spectra of PPTA-AM samples are presented in Figure 1. It can be observed that the absorption peaks around 1,640 and 1,545 cm^−1^ in PPTA fiber, which correspond to the C = O stretching vibration of amide and C-N stretching vibration, respectively (Yang et al., 2011), were weakened after modifications and appeared red-shifted in PPTA-AM samples. The peak at 1,574 cm^−1^ attributed to the in-plane bending vibration absorption of -NH_2_ (from APTES) (Mehdipouratae et al., 2013) was present in PPTA-PAPSQ (Wang et al., 2017) but absent in all PPTA-AM samples. This may be due to the interactions between the thiol groups in MPTES and the amino groups in APTES. Two broad and strong absorption peaks around 1,104 and 1,010 cm^−1^ in PPPTA-AM samples as compared to one intense absorption peak at about 1,130 cm^−1^ in both PPTA-A and PPTA-M samples, which can be attributed to –Si–O–Si– structure (Wang et al., 2017). This indicated that the structures of polysilsesquioxane with bifunctional groups of thiol- and amino- in PPTA-AM samples were different from those in mono-functional PPTA-A and PPTA-M adsorbents.

#### FE-SEM Images

**Figure 2:**
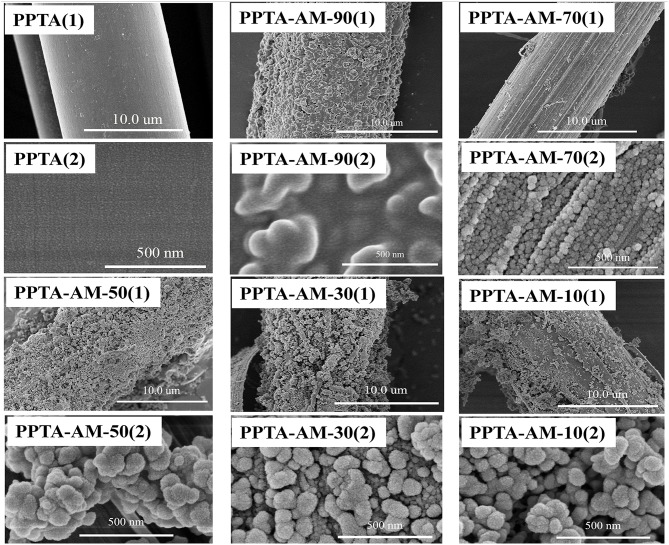
FE-SEM images of PPTA and PPTA-AM samples.

As shown in Figure 2, PPTA-AM samples coated with various amounts of polysilsesquioxane exhibited varied surface morphologies. PPTA-AM-90 possessed a compact porous surface coating, and the surface nanoparticles of polysilsesquioxane (NPPSQ) were tightly cemented together. Conversely, there were a large number of NPPSQs densely aggregated in the form of “pearl chains” on the surface of PPTA-AM-70. The NPPSQs were relatively evenly distributed on the surface of PPTA-AM-50 and PPTA-AM-30, but severely agglomerated on the surface of PPTA-AM-10. The above observations indicated that the ratio of APTES and MPTES had important effects on the morphology and structures of NPPSQs on the surface PPTA-AM samples. When the proportion of APTES was higher than that of MPTES, NPPSQs tended to form compact porous structures shown on PPTA-AM-90 and PPTA-AM-70, which was due to the hydrogen-bonding interaction of -NH_2_ in APTES (Wang et al., 2017). When the proportion of APTES were equal to (in the cases of PPTA-AM-50) or lower than that of MPTES (in the cases of PPTA-AM-30 and PPTA-AM-10), NPPSQs tended to form loose and porous structures because of the weakened hydrogen-bonding interaction of -NH_2_ caused by its interaction with –SH in MPTES.

#### XPS Analysis

**Figure 3:**
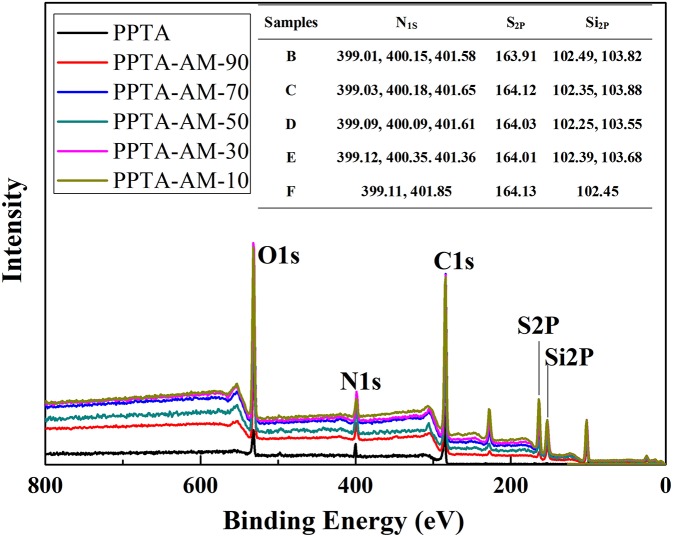
XPS spectra of PPTA and PPTA-AM samples.

XPS results of wide-scan spectra of PPTA and PPTA-AM are shown in Figure 3. The element concentrations (Table 1) of N showed a gradually decreasing trend while those of S showed a gradually increasing trend. However, the ratio of N to S on the surface of PPTA-AM was not the same as that in the reactant mixture of APTES and MPTES. The binding energies of N_1s_ showed three peaks of N_1S_ in PPTA-AM-90–PPTA-AM-30 that appeared at about 399, 400, and 401 eV, which can be assigned to NH_2_ (Metwalli et al., 2006; Majumder et al., 2009), amide (Giordani et al., 2009), and the protonated NH_2_ adjacent to Si-OH, respectively (Acres et al., 2012). The binding energies of Si_2p_ that appeared at about 102 and 103 eV indicated there were two types of Si in the polysilsesquioxane structure, which could be assigned to Si–O–Si and Si–OH, respectively (Kropman et al., 1997; Qiao et al., 2015). But in the spectrum of PPTA-AM-10, only one peak of Si_2p_ appeared at 102.45 eV, which was attributed to Si-O-Si due to the highest MPTES proportion. It should be noted that the binding energy of S_2P_ increased gradually from PPTA-AM-90 to PPTA-AM-10 due to the interaction between SH and NH_2_, indicating that the ratio of APTES and MPTES may have contributed to the difference of polysilsesquioxane structure in the PPTA-AM surface.

#### XRD Analysis and Nitrogen Adsorption Measurements

**Figure 4:**
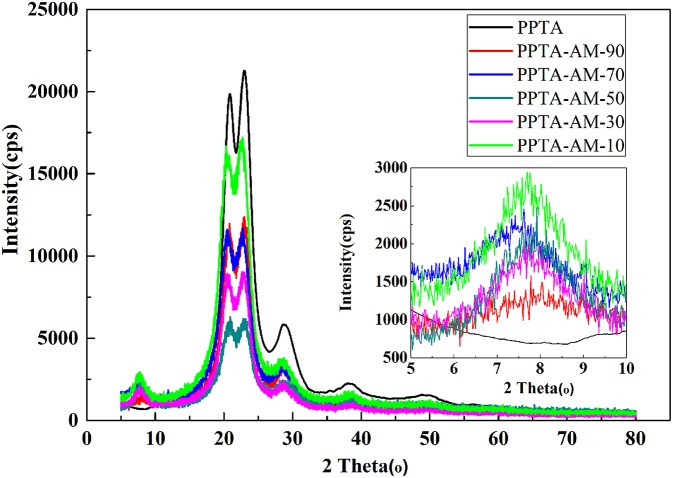
XRD patterns of PPTA and PPTA-AM samples.

The WAXD patterns of PPTA and PPTA-AM samples and their corresponding crystalline parameters are presented in Figure 4 and Table 2, respectively. As shown in Table 2, the Bragg angle (2θ) of (110), (200), and (211) crystal planes in PPTA-AM samples, slightly decreased compared with those of PPTA fibers, implying that the inter-planar spacing of each plane increased and the stacking density of the crystallite decreased (Zhang et al., 2006). Meanwhile, the values of full-width at half maximum (fwhm) and the average sizes of crystallites perpendicular to their diffracting planes (L_hkl_) of modified PPTA fibers (PPTA-AM) slightly increased. All the results suggested that the crystal structures of PPTA fibers were affected significantly after modifications, so that their relative crystallinity (crystalline index, CI) was decreased. The CI of PPTA-AM samples were lower than those of PPTA-A and closer to those of PPTA-M. This may be due to the weakened effect of NH_2_ on the crystal structure of PPTA fibers caused by the interaction between NH_2_ and SH. Similarly to findings in our previous work, the intensities of the diffraction peaks at 20.5 and 22.6° in PPTA-AM samples decreased compared with those of the PPTA fibers. This indicated the amorphous structures of polysilsesquioxane with thiol and amino of bifunctional groups on the surface of PPTA-AM (Li et al., 2015).

**Table 2:** Formulations, element concentrations, parameters of porous structures and ratio of functional group to metal ionon the surfaces of PPTA and PPTA-AM samples.

**Samples**	**BET surface area (m^**2**^ g^−1^)**	**BJH desorption cumulative pore volume (cm^**3**^ g^**−1**^)**	**BJH desorption pore diameter (nm)**	**2θ (**°**)**			**fwhm (**°**)**			**L**_**hkl**_ **(nm)**			**CI (%)**
				**(110)**	**(200)**	**(211)**	**(110)**	**(200)**	**(211)**	**(110)**	**(200)**	**(211)**	
PPTA	–	–	–	20.89	22.98	28.76	2.38	2.06	2.48	4.26	3.86	3.10	82.06
PPTA-AM-90	2.04	0.03	10~85	20.83	22.95	28.64	2.49	2.11	2.52	4.31	3.88	3.12	75.58
PPTA-AM-70	6.57	0.04	5~80	20.79	22.96	28.61	2.53	2.12	2.54	4.35	3.87	3.13	74.89
PPTA-AM-50	7.16	0.15	1~60	20.80	22.96	28.65	2.51	2.05	2.57	4.30	3.86	3.11	71.14
PPTA-AM-30	6.32	0.13	1~65	20.81	22.93	28.68	2.50	2.15	2.55	4.29	3.86	3.11	72.91
PPTA-AM-10	16.51	0.14	1~70	20.85	22.95	28.66	2.53	2.14	2.64	4.28	3.87	3.12	78.91

#### Nitrogen Adsorption Measurements

**Figure 5:**
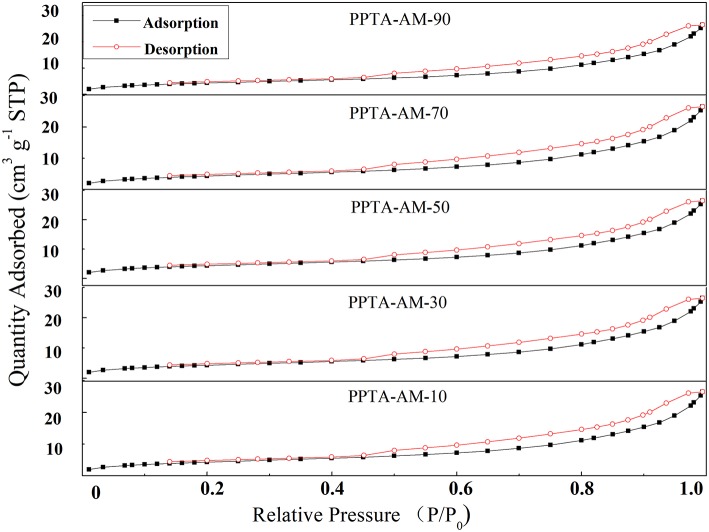
Nitrogen adsorption–desorption isotherms of PPTA-AM samples.

Results from the nitrogen adsorption-desorption experiments are shown in Figure 5. It can be seen that the curves of nitrogen adsorption -desorption isotherms PPTA-AM samples were classified as type IV with H3 type hysteresis loop according to the IUPAC classification (Zhao et al., 2012), suggesting that PPTA-AM samples contained meso- or macroporous structures. The hysteresis loops of all the PPTA-AM samples extrapolated almost to P/P0 = 1, suggesting complete filling of the mesopores (Adam et al., 2010), which is also regarded as one of the characteristics of solids consisting of aggregates or agglomerate particles forming slit-shaped pores with non-uniform size and shape (Ahmed and Adam, 2007).

Table 2 showed that the BJH desorption pore size distributions of PPTA-AM-90–PPTA-AM-10 were predominately in the ranges of 10~85, 5~80, 5~60, 1~65, and 1~70 nm, respectively, which were slightly lower than that of PPTA- M (5–107 nm). The above results demonstrated that the adsorbents with porous structures can be obtained by using the DRSF method.

### Saturated Adsorption

**Figure 6:**
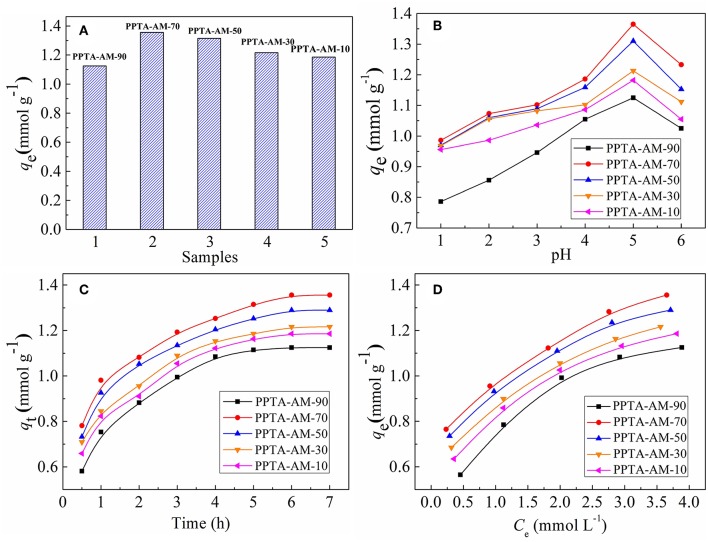
Saturated adsorption capacities **(A)**, effect of pH **(B)**, adsorption kinetics **(C)**, and adsorption isotherms for Hg(II) onto PPTA-AM samples **(D)**.

The saturated adsorption capacities of PPTA-AM for Hg(II) are presented in Figure 6A. For PPTA-A obtained using different quantity of APTES, the amounts of NH_2_ are 0.63, 0.99, 1.31, 1.51, and 1.52, and the adsorption amounts are 0.07, 0.20, 0.26, 0.28, and 0.28 mmol g^−1^. And for PPTA-M obtained using different quantity of MPTES, the amounts of SH are 1.41, 2.04, 2.18, 3.45, and 3.51 mmol g^−1^, and the adsorption amounts are 0.32, 0.58, 0.65, 0.78, and 0.78 mmol g^−1^. As shown in Figure 6A, the saturated adsorption capacities of PPTA-AM-90–PPTA-AM-10 for Hg(II) were 1.13, 1.36, 1.32, 1.22, and 1.19 mmol g^−1^, respectively. That is, anyone is much higher than that adsorption maximum of PPTA-A (0.28 mmol g^−1^, with the highest amount of NH_2_ is 1.52 mmol g^−1^) and that adsorption maximum of PPTA-M (0.78 mmol g^−1^, with the highest amount of SH is 3.51 mmol g^−1^). Obviously, the adsorption effect of amino- and thiol- polysilsesquioxane simultaneously coating on poly(*p*-phenylenetherephthal amide) fibers were much better than mono-functionalized poly(p-phenylenetherephthal amide) fibers (PPTA-A and PPTA-M) befitting from the synergistic effect. The adsorption capacity of PPTA-A was lower than those of PPTA-AM, which could be attributed to the lower loading of polysilsesquioxanes coating on the surface. The only explanation for the much higher adsorption capacities of PPTA-AM is that the polysilsesquioxanes with bifunctional groups in PPTA-AM samples were of different form and more conducive to Hg(II) adsorption than those in PPTA-M. Meanwhile, the Hg(II) adsorption capacities of PPTA-AM followed the descending order of PPTA-AM-70, PPTA-AMPPTA-AM-50, PPTA-AM-90, PPTA-AM-30, and PPTA-AM-10. This may be caused by a variety of factors such as the content of functional groups, pore structure and morphology of polysilsesquioxanes coating.

### Determination of the Optimum pH Value

The pH value of the metal ions solution can impact the interaction between metal ions and the surface structure of adsorbents. The relationship between the saturated adsorption capacities and pH is shown in Figure 6B. It was found that the maximum adsorption values for the Hg(II) onto PPTA-AM samples appeared at pH = 5.0. At low pH, NH_2_, and SH on the surface of adsorbents were positively charged owing to protonation, thus the adsorption was dominated by electrostatic repulsion and disfavored. Moreover, the existence of a large amount of H^+^ in the solution may compete adsorption with Hg(II). With the increase of pH, the electrostatic repulsion between PPTA-AM and Hg(II) reduced for deprotonation and the deprotonated NH_2_ and SH can effectively coordinate with Hg(II), thus the adsorption capacity of PPTA-AM for Hg(II) increased (Ma et al., 2017). The uptake of Hg(II) beyond pH 5.0 is decreased, which might attribute the formation of metal hydroxide species such as soluble Hg(OH)^+^. Therefore, the optimum value pH = 5.0 was chosen in the subsequent experiments. This value was the same for PPTA-A and PPTA-M, implying that the interaction of NH_2_ and SH did not affect the optimum pH values.

### Adsorption Kinetics and Adsorption Isotherms

The adsorption kinetics for Hg(II) of PPTA-AM were determined to investigate the adsorption behavior and the results are shown in Figure 6C. As shown in Figure 6C, the adsorption for Hg(II) of PPTA-AM samples that reached equilibrium required a minimum of 6 h. The experimental data were tested by using to Pseudo-first-order (Barkat et al., 2009) and pseudo-second-order (Ho et al., 2000) models and the adsorption kinetic process were thus elucidated. The adsorption kinetics for Hg(II) of PPTA-AM were better described by pseudo-second-order model and equilibrium required a minimum within 6 h as shown in Figure 6B. The adsorption rate and adsorption capacities of PPTA-AM-70 were maximum, due to porous structure and to proceed the bicontinuum process, which occurred either in series or in parallel, being the more common bicontinuum conceptualization (Brusseau et al., 1989).

The pseudo-first-order and pseudo-second order models are, respectively, expressed by Equations (2) and (3).

(2) $$\begin{array}{l} \ln\;\left(q_{e}-q_{t}\right)=\ln\;q_{e}-k_{1}t \end{array}$$

(3) $$\begin{array}{l} \frac{t}{q_{t}}=\frac{1}{k_{2}q_{e}^{2}}+\frac{t}{q_{e}} \end{array}$$

*q*_e_ and *q*_t_ (mmol g^−1^) are the adsorption amounts of Hg(II) at equilibrium and time *t* (min), respectively. *k*_1_ and *k*_2_ are the rate constants of pseudo-first-order (h^−1^) and pseudo-second-order (g mmol^−1^ h^−1^) adsorption. The experimental and calculated *q*_e_ values, rate constants and regression coefficient (R^2^) values are all presented in Table 3.

**Table 3:** Kinetic parameters for the adsorption of Hg(II) on PPTA-AM samples.

Adsorbents	*q_*exp*_* (mmol g^−1^)	Pseudo-first-order kinetics			Pseudo-second-order kinetics		
		*k_1_* (h^−1^)	*q_*e*(*cal*)_* (mmol g^−1^)	$R_{1}^{2}$	*k_2_* (g mmol^−1^ h^−1^)	*q_*e*_* (mmol g^−1^)	$R_{2}^{2}$
PPTA-AM-90	1.125	0.156	0.858	0.9412	1.579	1.118	0.9972
PPTA-AM-70	1.365	0.197	1.154	0.9695	1.891	1.356	0.9973
PPTA-AM-50	1.315	0.183	0.109	0.9754	1.645	1.321	0.9975
PPTA-AM-30	1.216	0.173	0.965	0.9855	1.441	1.210	0.9973
PPTA-AM-10	1.186	0.160	0.956	0.9653	1.370	1.185	0.9962

As shown in Table 3, the adsorption process followed the pseudo-second-order model well and had better correlation coefficients than the pseudo-first-order model for samples. Therefore, the adsorption kinetics of Hg(II) onto the adsorbents was better described by pseudo-second-order model.

The isotherm adsorption of PPTA-AM for Hg(II) was investigated and the results are shown in Figure 6D. The Langmuir and Freundlich equations were adopted to fit the experimental data. The linear expressions of Langmuir and Freundlich models can be written as Equations (4) and (5) (Zhang et al., 2015):

(4) $$\begin{array}{l} \frac{C_{e}}{q_{e}}=\frac{C_{e}}{q}+\frac{1}{qK_{L}} \end{array}$$

(5) $$\begin{array}{l} \ln\;q_{e}=\ln\;K_{F}+\frac{\ln\;C_{e}}{n} \end{array}$$

where *q*_e_ is the equilibrium concentration of Hg(II) on the adsorbent (mg g^−1^), *C*_e_ is the equilibrium concentration of Hg(II) in solution (mg L^−1^), *q* is the maximum capacity of adsorbent (mg g^−1^), and *K*_L_ is the Langmuir adsorption constant (L mg^−1^). *K*_F_ is the binding energy constant reflecting the affinity of the adsorbents to metal ions; *n* is the Freundlich exponent related to adsorption intensity.

The corresponding Langmuir and Freundlich constants and correlation coefficients (R^2^) are listed in Table 4. From the correlation coefficients in Table 4, it can be concluded that the experiment data fitted Langmuir equation better than Freundlich equation, revealing the adsorption of Hg(II) adsorption on PPTA-AM obeyed the Langmuir adsorption isotherm. This implies that the adsorption of Hg(II) on PPTA-AM followed the mechanism of monolayer adsorption (chemisorption) (Qu et al., 2013).

**Table 4:** Langmuir and Freundlich isotherm constants for the adsorption of Hg(II) on PPTA-AM at 25°C (pH 5.0).

Adsorbents	Langmuir			Freundlich			Ratio of functional group to metal ion^*^
	*q*_the_ (mmol g^−1^)	*K*_L_ (L mmol^−1^)	$R_{L}^{2}$	*K_*F*_* (mmol g^−1^)	*n*	$R_{F}^{2}$	(NH_2_+SH)/Hg(II)
PPTA-AM-90	1.32	1.42	0.9982	0.051	1.80	0.9802	1.36
PPTA-AM-70	1.54	0.41	0.9961	0.10	2.42	0.9775	1.25
PPTA-AM-50	1.41	0.43	0.9914	0.11	1.98	0.9682	1.42
PPTA-AM-30	1.34	0.45	0.9912	0.15	1.68	0.9743	1.49
PPTA-AM-10	1.33	0.54	0.9941	0.041	1.57	0.9891	1.42

* *The Ratio of functional group to metal ion is equal to M/q; wherein, M presents the amount of functional group (NH_2_ + SH) in PPTA-AM (Table 1)*.

Based on the q_*the*_ values in Table 4, these values have a significant advantage over other silica adsorption materials, e.g., silica–dithizone at 0.22 mmol g^−1^ (Cestari et al., 2004), and pure functionalized polysilsesquioxane, e.g., diethylenetriamine-bridged polysilsesquioxanes at 1.81 mmol g^−1^ (Sun et al., 2014), POSS-SH at 0.06 mmol g^−1^ (Wang et al., 2014). They are also higher than those of the monofuctional polysilsesquioxanes coated PPTA fibers, e.g., PPTA-A at 10.64 mmol g^−1^ and PPTA-M at 10.22 mmol g^−1^ (Wang et al., 2017). This implies that PPTA-AM with amino- and thiol- bifunctional groups possessed higher functional group utilization rates of polysilsesquioxanes than PPTA-A and PPTA-M adsorbents with amino- or thiol- monofunctional groups.

### Adsorption Selectivity

The adsorption selectivities of PPTA-AM-70 were chosen as representatives in binary ion systems to compare the differences in adsorption properties between adsorbents with bifunctional groups and those with monofunctional groups. The results are presented in Table 5. From Table 5, it was found that PPTA-AM-70 exhibited excellent selectivity toward Hg(II) in the presence of Pb(II), Cu(II), Ni(II), and Cd(II), implying that bifunctionalization had no significant effect on its adsorption selectivity.

**Table 5:** Adsorption selectivity of PPTA-AM-70 toward Hg(II) at 25°C (pH 5.0).

**System**	**Mental ions**	***q* (mmol g^**−1**^)**	Selectivity coefficient^*^
Hg(II)-Pb(II)	Hg(II)	1.36	∞
	Pb(II)	0.00	
Hg(II)-Cu(II)	Hg(II)	1.35	∞
	Cu(II)	0.00	
Hg(II)-Ni(II)	Hg(II)	1.36	∞
	Ni(II)	0.00	
Hg(II)-Cd(II)	Hg(II)	1.36	∞
	Zn(II)	0.00	
Hg(II)-Ag(I)	Hg(II)	1.31	10
	Ag(I)	0.13	

* *The selective coefficient was the ratio of adsorption capacities of metal ions in a binary system*.

### Adsorption Mechanism

**Figure 7:**
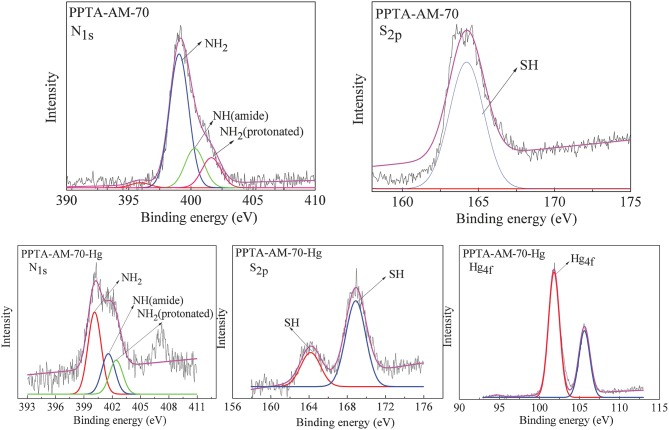
N_1s_, S_2p_, Hg_4f_ spectra of PPTA-AM-70 after Hg(II) adsorption.

The adsorption mechanism of PPTA-AM for Hg(II) using the XPS technique by comparing the changes of binding energies of N_1s_, S_2p_, and Hg_4f_ before and after adsorption. Figure 7 shows the N_1s_, S_2p_, and Hg_4f_ spectra of PPTA-AM-70 after adsorbing Hg(II). From Figure 7, it can be found that the binding energies of N_1s_ in NH_2_ and S_2p_ in SH in PPTA-AM-70 were shifted from 399.03 to 399.68 eV and from 164.12 to 164.32 eV, respectively, while that of Hg_4f_ was shifted from 104.00 to 101.98 eV after adsorption, implying that both NH_2_ and SH were involved in the coordination with Hg(II). The binding energy of N_1s_ of amide 400.18 eV in PPTA-AM-70 had no change before and after adsorption, indicating that N of amide was not involved in adsorption process. The binding energy of S_2p_ at 168.32 eV implied that there was a certain degree of redox reaction in the adsorption process (Qu et al., 2006).

**Scheme 2:**
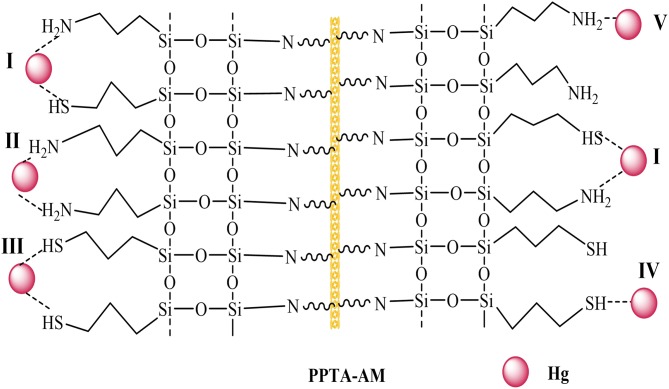
Possible chelating structures of PPTA-AM with Hg(II).

According to the data in Table 4, the coordination ratio of (NH_2_ + SH) to Hg(II) of PPTA-AM samples can be calculated to be 1.36, 1.25, 1.42, 1.49, and 1.42, respectively, which were much lower than 2.4–2.89 of NH_2_/Hg(II) for PPTA-A and 2.56–3.96 of SH/Hg(II) for PPTA-M ones. This suggested that PPTA-AM adsorbents needed only 1.25–1.49 (NH_2_+SH) to chelate one Hg(II) ion. In other words, the bifunctional adsorbents prepared by the DRSF method had higher functional group utilization than those with monofunctional groups. This may be a result of their unique loose open meso- or macroporous features of PAMPAQ. Based on the above analysis, the adsorption mechanism of the PPTA-AM samples is proposed as illustrated in Scheme 2. The structures of chelates depended on the proportion of NH_2_ and SH in PAMPSQ. When the proportion of NH_2_ was greater than SH, the structure was dominated by I, II and IV. When the proportion of NH_2_ was lower than SH, the structure was dominated by I, III, and V. When the proportion of NH_2_ was equal to SH, the structure was dominated by I, IV, and V.

### Regeneration

PPTA-AM-70 was chosen as a representative to test the reusability. The elution rate was over 95% eluted by the eluent system of 4% thiourea in 0.5 M HCl. Thiourea in HCl is frequently used for the desorption of Hg(II) from adsorbent surfaces because the sulfur and nitrogen present in thiourea can form a coordination interaction with Hg(II) ions, and because thiourea dissolved in HCl is expected to have more of a desorption effect (Velempini et al., 2019). Five cycles of adsorption-desorption were carried out on a single adsorbent sample and the results are shown in Table 6. As can be seen from Table 6, the adsorption properties of PPTA-AM-70 had only a small decrease after five cycles of adsorption-desorption, with the uptake all being above 90%. Therefore, the adsorbent was suitable for repeated use at a diminishing rate in the adsorption capacity.

**Table 6:** Regeneration properties of PPTA-AM-70 for Hg(II) adsorption.

**Regeneration times**	**Q_**e**_ (mmol g^**−1**^)**	**Desorption rate (%)**
1	1.365	94.52
2	1.355	93.53
3	1.341	92.23
4	1.325	90.56
5	1.306	90.55

## Conclusion

Bifunctional adsorbents PPTA-AM, amino- and thiol- polysilsesquioxane (PAMPSQ) simultaneously coated poly(*p*-phenylenetherephthal amide) fibers were successfully prepared by developed DRSF method. The loose and meso- or macro-porous structures of PAMPSQs in the form of nanoparticles were formed on the surface of PPTA fibers. The morphologies of the PAMPSQ coatings were dependent on the the proportion of reactants of APTES and MPTES. PPTA-AM adsorbents just need 1.25–1.49 NH_2_+SH chelate one Hg(II) ion, indicating that the PAMPSQ coating in these bifunctionals adsorbents had much higher functional group utilization than pure functionalized polysilsesquioxane adsorption materials, and the corresponding monofunctional polysilsesquioxane coated PPTA fibrous adsorbents for Hg(II) adsorption.

## Data Availability

The datasets generated for this study are available on request to the corresponding author.

## Author Contributions

RQ: work design. YW: data collection and organize the draft. YM, KA, XJ, and YuZ: data collection. CS and CJ: characterization. YiZ: make important changes to the paper.

### Conflict of Interest Statement

The authors declare that the research was conducted in the absence of any commercial or financial relationships that could be construed as a potential conflict of interest.
